# A blueprint for the next generation of ELSI research, training, and outreach in regenerative medicine

**DOI:** 10.1038/s41536-017-0026-z

**Published:** 2017-07-05

**Authors:** Judy Illes, Douglas Sipp, Erika Kleiderman, Shelly Benjaminy, Rosario Isasi, Geoff Lomax, Zubin Master, Jennifer McCormick, Ubaka Ogbogu, Vardit Ravitsky, Julie M. Robillard, Fabio Rossi, Brenda Wilson, Amy Zarzeczny

**Affiliations:** 1 National Core for Neuroethics, Division of Neurology, Department of Medicine, University of British Columbia, Vancouver, BC Canada; 2 RIKEN Center for Developmental Biology, Kobe, Japan; 3 Keio University School of Medicine, Tokyo, Japan; 4 Keio Global Research Institute, Tokyo, Japan; 5 Centre of Genomics and Policy, McGill University, Montreal, QC Canada; 6 National Core for Neuroethics and Department of Experimental Medicine, University of British Columbia, Vancouver, BC Canada; 7 John T. Macdonald Foundation Department of Human Genetics, University of Miami, Coral Gables, FL USA; 8 California Institute of Regenerative Medicine, Oakland, CA USA; 9 Alden March Bioethics Institute, Albany Medical College, Albany, NY USA; 10 Department of Humanities, Pennsylvania State College of Medicine, Hershey, PA USA; 11 Faculties of Law and Pharmacy & Pharmaceutical Sciences, University of Alberta, Edmonton, AB Canada; 12 Bioethics Program, School of Public Health, University of Montreal, Montreal, QC Canada; 13 Department of Medical Genetics, University of British Columbia, Vancouver, BC Canada; 14 School of Epidemiology, Public Health and Preventive Medicine, University of Ottawa, Ottawa, ON Canada; 15 Johnson Shoyama Graduate School of Public Policy, University of Regina, Regina, SK Canada

## Abstract

Regenerative medicine has attracted the interest of scientists, physicians, and patient communities, and as well as policy-makers and the broader public given related ethical, legal, and social implications. Here we examine past initiatives in the ethical, legal and social implications arena in regenerative medicine, and offer our views on actionable priorities for the future in six key areas: capacity building, policy, engagement with industry, resaerch ethics, communication, and community building.

## Building on the past and anticipating the future

Research and development (R&D) in the sphere of advanced biomedical technologies involves dialog and negotiations across a spectrum of stakeholders with diverse interests. Nowhere is this seen more clearly than in regenerative medicine, which has attracted not only the interest of scientists, physicians and patient communities but, due its to related ethical, legal, and social implications (ELSI), that of the broader public and policy-makers. In response, countries home to significant initiatives in the development of regenerative medicine technologies (Table 1) have also invested in studies to illuminate understandings and tensions that the field raises, mitigate potential risks, and facilitate the translation of research products into the clinic.

**Table 1:** Key Terms and Concepts

Term	Definition
Regenerative Medicine	A field of clinical research and application that seeks to use biological materials and mechanisms to repair, restore, maintain or improve the function of tissues and whole organs
Stem cells	Cells with the ability to self-renew and to give rise to progeny cells of different types. Stem cells are often categorized by their developmental potential (e.g., pluripotency or multipotency) or by tissue source. These cells play important roles in development, tissue homeostasis, and regeneration. Examples are:
Embryonic stem cells (ESCs)	Cells derived from the inner cell mass of the blastocyst, which can be induced to give rise to cells representing all three germ layers in vitro, the primary hallmark of pluripotency
Induced pluripotent stem cells (iPSCs)	Somatic cells reprogrammed to a state of pluripotency, typically using a combination of transcription factors introduced via a viral vector or other method
Tissue stem cells	Also sometimes referred to as ‘somatic’ or ‘adult’ stem cells, these cells are present in various tissues in the adult body. They typically show a much more limited range of lineage-specific differentiation, known as multipotency
Progenitor cells	Cells that originated during the differentiation process of a stem cell. They are tissue-specific in nature and lack self-renewal capacity
Gene editing	Techniques for deleting, adding or otherwise manipulating genomic DNA sequences in both somatic and germline cells in many species. Common technologies include zinc-finger nucleases, TALENs, and CRISPR/Cas9
Biobanks	Biorepositories for storing data and biological samples for research and/or clinical purposes. The governance structure of most biobanks establishes defined procedures for access to biological samples and data
Tissue engineering	Biorepositories for storing data and biological samples for research and/or clinical purposes. The governance structure of most biobanks establishes defined procedures for access to biological samples and data

Canada has shown consistent leadership in its national efforts to promote impactful ELSI research into regenerative medicine, such as stem cell research, tissue engineering, and gene editing^1, 2^. For example, since its launch in 2001, the $90 M CAD Stem Cell Network (SCN), has been a catalyst for the translation of stem cell research into clinical and commercial products, and for innovative programs in ethics, law, sociology, health economics, communications, and other societal developments in this rapidly evolving area^3^. Early successes included the establishment of a global policy database of regulations on somatic cell nuclear transfer and human embryonic stem cell research, legal scholarship into intellectual property, biobanking, confidentiality, consent, and extensive public outreach, and partnerships with organizations internationally^4^. However, the rapid pace of scientific change, with fundamental discoveries including induced pluripotent stem cells (iPSCs) and targeted genome editing using novel techniques such as CRISPR/Cas9, has resulted in a shifting landscape of expectations. Here we discuss how the Canadian and global ELSI communities can respond to this shift and promote sustainable, socially-minded advances in regenerative medicine.

## Capacity building across a range of careers

Training is a cornerstone of regenerative medicine; today’s trainees will be tomorrow’s leaders and innovators. Opportunities for observation and immersive experience are critically important to informing the conceptual and empirical contributions of scholars working at the nexus of science and society. Creating pathways to bring researchers from wet labs together with their dry lab counterparts who study issues in ethics, law, and other social sciences will deliver key benefits to both groups. To this end, in Canada, we are exploring the launch of a scientist-society series for trainees to gain first-hand experience working within a research group outside their core field. In this model, ELSI trainees are exposed to biomedical research as it is practiced, with all the advantages, limitations, and timeframes around the development of technology or therapeutic strategies. Reciprocally, for laboratory scientists, time embedded within the ELSI world is designed to broaden perspectives into the profound and sometimes under-appreciated human tensions surrounding cutting-edge research.

Much still remains to be learned about how scientists—whether young or seasoned—perceive ethics, law, and social issues, what they expect from ELSI research, and the extent to which studies impact their own research. Social science approaches are ideally suited for identifying and elaborating scientists’ perceptions of ELSI, as well as the inverse: informing social scientists’ perspectives of scientists’ practices and responsible conduct of research. Public and private sector grants will be essential to power efforts that bridge these important knowledge gaps.

## Shaping the policy landscape

Rapid advances in biomedical R&D over the past decade have often forced policy-makers to play catch-up. The advent of iPSCs not only transformed the understanding of cellular differentiation, but also loosened the deep connections between pluripotency and thorny ethical questions that mandated strict governance. Regenerative medicine policies in Canada and other countries are thus ripe for review and, in some cases, reform. In this regard, we recommend that leaders in the international regenerative medicine community, such as the SCN, the International Society for Stem Cell Research, the International Society for Cellular Therapy, and the California Institute of Regenerative Medicine among others, undertake a broad survey of relevant laws, regulations and guidelines relating to the R&D of regenerative medicine and related biomedical technologies, issue policy statements, and consider action in reforming extant policies. The 14-day restriction on embryo research that has been revisited and is a source of emergent discussion is one example^5^. In addition to reviewing existing administrative and civil laws, leading organizations need to better engage with disease advocacy organizations and identifiable well-organized patient groups to explore and jointly articulate how regulations impact patients and can better serve their needs. Confidentiality of personal information, including genetic information, and consent to uses of donated biological materials remain two centrally divisive issues in the area of biobanking and clinical research^6–10^. Importantly, different communities with similar end goals may hold divergent views about sharing personal information for research purposes, the obligation to inform donors of incidental findings, or the degree to which donor consent can be extended to unanticipated uses^11^.

Approval pathways for cell-based, tissue-based, and gene-based therapeutics have also been evolving in remarkable ways. Recent years have seen the de facto relaxation of efficacy standards for cell products in South Korea, followed by de jure deregulation in Japan^12^. Such surveillance work is particularly important to regenerative medicine, a field in which the regulatory and policy landscape is fragmented and rapidly evolving away from a uniform global standard^13^. In the USA, recent efforts have exposed an appetite for access to investigational products with the Right to Try laws. The newly elected Trump administration has signaled that it favours radical deregulation of the national health market as well^14^.

Confronted with the realities of economic competition, other countries are taking notice. A patient-centric analysis of the arguments for the benefits of paced development will have to be balanced against a comprehensive account of the costs to society through lost therapeutic opportunity in time, money, and human resource efforts^15^. Advocates of deregulation have been quick to equate shortcuts that are economically advantageous for the private sector with benefits to patients. However, the long history of US FDA and its experiences in protecting the public from unsafe or ineffective medicines indicates that this optimism may be ill-placed. A critically-minded, independent investigation of these questions is needed to inform national policy in ways that serve the public need for safe, effective, and valuable regenerative medicine.

## Industry engagement

Research ethics calls for careful management of potential conflicts of interest when engaging with industry, yet there is urgency for greater learning about the demands and constraints faced by industry and for mutual problem solving. One approach to respond to this imperative is to incentivize the integration of ELSI research components into research using human biomaterials and clinical trials. We suggest, for example, that including ELSI researchers as co-investigators on grant-funded studies would add an important new dimension to them. This might involve, for example, a genetic counselor who studies donor consent for use of genetic materials, an ethicist interested in issues of access to an early clinical trial using a novel tissue engineering approach and who can provide frameworks or logic models for decision-making, or a health economist evaluating an investigational product with an anticipated high reimbursement profile. Budgetary weight should be proportional to the costs of the research and might therefore be disproportional overall, and we emphasize that it is important to decouple dollars from value contributions.

## Evolving practices in research ethics

Core principles articulated in Canada’s *Tri-Council Policy Statement* and other guidance are essential to the design and conduct of research that meets high scientific and ethical standards, and respects and protects participants. As for any best practice, continuous reassessment is vital^16^. To support adherence to the core principles, related desiderata about research integrity, accountability, reproducibility, transparency, and public trust, we emphasize the inclusion of stakeholders, especially end-users such as patients and their advocates in the design of studies involving human subjects or biomaterials. Such practices should encompass underserved groups, including persons affected by rare diseases, as well as culturally diverse communities. In many countries, including Canada, the voice of and values of Indigenous Peoples regarding regenerative medicine must be considered in such frameworks. Mechanisms for handling donor genetic data and private information, consent, resource use, and access are all issues best evaluated at the time of study design, rather than retrospectively or reactively.

## Communication

Traditional and social media play a crucial role in filtering, sharing, and interpreting science and policy for the public sphere. These roles can be positive when they build awareness, interest, and understanding by delivering high quality information in accessible formats and by encouraging dialog. When they distort or exaggerate, however, media serve neither the public interest nor the interests of scientific progress. The ELSI community can and should play a role both by studying the impact of these new modes of popular media and engaging directly with the broader public and patient communities through participation in virtual communities and networks. This effort should not only to point out misinformation or ethical concerns, but also serve to highlight new opportunities for the field to better integrate public and patient perspectives and interact with society.

Researchers have performed valuable analyses of depictions of stem cells, for example, and human genetics in popular media and in online social networking^17–19^. Individual scholars have also served an important corrective function by challenging inaccurate portrayals in areas such as unproven cell-based interventions, human-animal chimera research, and near-term prospects for human gene editing^20^. Public education efforts, with a special focus on patients on the one hand, and on students on the other, are invaluable in cultivating understanding and curiosity about the hopes, promises, and limitations of regenerative medicine in our society.

## Community-building

Capacity development, policy work, stakeholder engagement, public communications, and development of best practices rely on the continued growth of a vibrant and interactive ELSI community. Investments in risky, blue-sky approaches for cross-pillar interactions are essential. Equally important is the implementation of tangible, quantifiable performance indicators assessing the impact of all evidence-based initiatives, including:

- advances in collaborative research projects,
- the ability of stem cell scientists and ethics scholars to implement multidisciplinary skills in their professional lives,
- educational ELSI initiatives to train future generations of scientists and scholars and,
- strengthened local capacity via implementation of research ethics governance bodies, and uptake of ethics policies that are distinct from systems already in place.

Individual scholars will continue to engage with the world through active participation in international initiatives, and through the leading work for which the Canadian and other ELSI communities are known.
